# Seroprevalence of avian influenza A (H5N1) virus among poultry workers in Jiangsu Province, China: an observational study

**DOI:** 10.1186/1471-2334-12-93

**Published:** 2012-04-18

**Authors:** Xiang Huo, Rongqiang Zu, Xian Qi, Yuanfang Qin, Liang Li, Fenyang Tang, Zhibin Hu, Fengcai Zhu

**Affiliations:** 1 Jiangsu Provincial Center for Disease Control and Prevention, Jiangsu Rd. 172#, Nanjing 210009, China; 2 College of Public Health, Nanjing Medical University, Hanzhong Rd. 140#, Nanjing, China

**Keywords:** Avian influenza, H5N1, Seroprevalence, Risk factors, Subclinical infection

## Abstract

**Background:**

Since 2003 to 06 Jan 2012, the number of laboratory confirmed human cases of infection with avian influenza in China was 41 and 27 were fatal. However, the official estimate of the H5N1 case-fatality rate has been described by some as an over estimation since there may be numerous undetected asymptomatic/mild cases of H5N1 infection. This study was conducted to better understand the real infection rate and evaluate the potential risk factors for the zoonotic spread of H5N1 viruses to humans.

**Methods:**

A seroepidemiological survey was conducted in poultry workers, a group expected to have the highest level of exposure to H5N1-infected birds, from 3 counties with habitat lakes of wildfowl in Jiangsu province, China. Serum specimens were collected from 306 participants for H5N1 serological test. All participants were interviewed to collect information about poultry exposures.

**Results:**

The overall seropositive rate was 2.61% for H5N1 antibodies. The poultry number was found associated with a 2.39-fold significantly increased subclinical infection risk after adjusted with age and gender.

**Conclusions:**

Avian-to -human transmission of avian H5N1 virus remained low. Workers associated with raising larger poultry flocks have a higher risk on seroconversion.

## Background

Since 2003 to 06 Jan 2012, the number of laboratory confirmed human cases of infection with avian influenza worldwide was 576 and 339 were fatal. In China, the number was 41 and 27 were fatal [1]. In December 2007, a family cluster of 2 individuals infected with highly pathogenic avian influenza (HPAI) A (H5N1) virus was identified in Jiangsu Province, China. One case died [2]. However, the official estimate of the H5N1 case-fatality rate has been described by some as an over estimation since there may be numerous undetected asymptomatic/mild cases of H5N1 infection [3]. One particularly important hypothesis is that cases are being missed because current surveillance primarily detects severe infections [4]. This study was designed to better understand the real infection rate of H5N1. It investigated the potential for the zoonotic spread to humans and evaluates the risk factors associated with subclinical infection among poultry workers in Jiangsu, China.

## Methods

### Samples and data collection

3 villages nearby habitat lakes of wildfowl were selected randomly in Gaochun, Jianhu and Gaoyou counties respectively in Jiangsu Province, China. From 1 July through 15 August 2010, we interviewed all the poultry workers from backyard poultry farms in these 3 villages to collect demographic data and information about poultry exposure. In addition, 5 mL of blood was collected for antibody test. All the poultry workers reported no serious respiratory illness history and their job duties were similar, including feeding poultry, collecting eggs and cleaning poultry stalls. Poultry from backyard farms were not vaccinated against avian influenza. Written informed consents were received from all participants. The study was approved by the Ethics Committee of Jiangsu Provincial Center for Disease Control and Prevention.

### Hemagglutination inhibition (HI) assay

All sera were tested by haemagglutination inhibition (HI) assay to detect H5-specific antibody as previously described [5,6]. Horse red blood cells were used since horse RBC HI assay has high reliability and good agreement with MN assay results [7]. Jiangsu Provincial Center for Disease Control and Prevention is one of the authorized national level influenza surveillance laboratory. China Center for Disease Control and Prevention, WHO (World Health Organization) reference laboratory, provided the reference antigens of A/Anhui/1/05 and A/Hubei/1/10 and the experiment training. In brief, the sera were treated with RDE by diluting one part serum with three parts enzyme and were incubated overnight in a 37°C water bath. The enzyme was inactivated by 30-min incubation at 56°C followed by addition of six parts 0.85% physiological saline for a final dilution of 1/10. According to WHO recommendations, a serum sample was considered as positive when the HI test antibody titer was ≥1:160 [8].

### Statistical analysis

Kolmogorov-Smirnov test was used to test the normality of data. We compared proportions/rates with use of Continuity Correction Chi-Square Test/Kruskal Wallis test, and median values with use of the Kruskal Wallis test. The 95% confidence intervals (CIs) of positive rates were calculated using Binomial Exact test. In logistic regression analysis, maximum likelihood estimates for the odds ratios (ORs) and 95% confidence intervals (CIs) were calculated using Wald X^2 ^test. Spearman correlation coefficients were used to evaluate the correlation between poultry numbers and serum positive rates of poultry workers. All tests were 2-tailed; statistical significance was set at *P *≤ 0.05. All the statistical analysis was performed with Statistical Analysis System software (9.1.3; SAS Institute, Cary, NC, USA).

## Results

A total of 306 serum samples were collected and tested. One hundred samples were from Gaochun, 76 samples from Jianhu and 130 samples from Gaoyou. The median and quartiles of ages of poultry workers were 58.00 (45.75-64.25). The sex ratio of workers was 1:1.59. All the serum samples were negative for antigen of A/Hubei/1/10. Thus the test results for antigen of A/Anhui/1/05 were used for analyses. The overall seropositive rate of H5N1 was 2.61% (95%CI, 1.14%-5.09%) in this survey. The positive rates of samples collected from Gaochun, Jianhu and Gaoyou were 0, 1.32% (95%CI, 0.03%-7.11%) and 5.38% (95%CI, 2.19%-10.78%), respectively, with significant difference (***P ***= 0.029, Kruskal-Wallis Test).

The median and quartiles of raising poultry numbers were also found significantly different among 3 counties (***P ***< 0.0001, Kruskal-Wallis Test). The numbers were 5.00 (3.00-7.00), 14.50 (7.25-24.50) and 18.00 (13.00-28.00) in Gaochun, Jianhu and Gaoyou, respectively.

**Figure 1:**
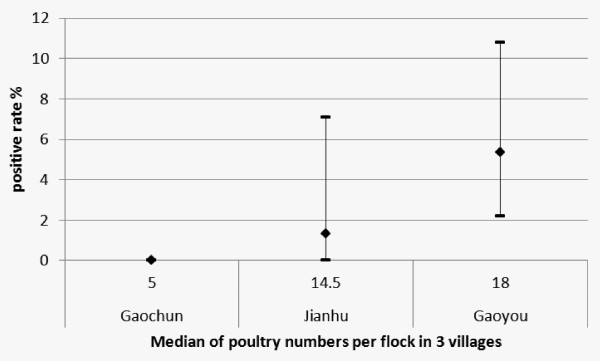
**Positive rates of H5N1 antibodies among poultry workers with various numbers of poultry per flock**. *The 95% confidence intervals (CIs) were calculated using Binomial Exact test.

In correlation analyses, the seropositive rates of H5N1 virus significantly correlate with the medians of raising poultry numbers (r = 1.00 and ***P ***< 0.01 for spearman test) (Figure 1). And the antibody titers against H5N1 virus also significantly correlate with the raising poultry numbers (r = 0.181, ***P ***= 0.001).

The distribution of gender and age between seropositive (HAI titers ≥ 1:160) and seronegative poultry workers was not significantly different (***P ***for gender = 0.667, Continuity Correction null Chi-Square Tests; ***P ***for age = 0.066, Kruskal-Wallis Test). The median of raising poultry number was significantly bigger in seropositive workers than in seronegative workers (***P ***= 0.026, Kruskal-Wallis Test). Unconditional logistic regression analysis was performed to evaluate the association of raising poultry numbers and risk of seropositive of PWs. The poultry number was found associated with a 2.39-fold increased risk after adjusted with age and gender (95%CI, 1.00-5.69) (Table 1).

**Table 1:** Distributions of gender, age and poultry number between seropositive and seronegative poultry workers (PWs)

Variables		Seronegative PWs	Seropositive PWs	*P*	OR(95%CI)
Gender	Male, no. (%)	116 (98.31)	2 (1.69)	0.667^a^	1.90 (0.37-9.85)
	Female, no. (%)	182 (96.81)	6 (3.19)		
Age	Median, quartiles	58.00 (45.00-64.00)	64.00 (61.25-69.25)	0.066^b^	1.04 (0.98-1.11)
Poultry Number	Median, quartiles	11.00 (6.00-20.00)	18.50 (14.50-29.50)	0.026^b^	2.39^c ^(1.00-5.69)

^a ^Continuity Correction Chi-Square Tests.

^b ^Kruskal-Wallis Test.

^c ^Poultry number was transmitted to ordinal variable using quartiles in the logistic regression model.

## Discussion

Several serological studies have been conducted to assess the transmission of H5N1 virus in poultry workers. The reported seropositive rates vary from 0 to 10% [9-11]. In the present study, the overall positive rate we found was 2.61% and in different sampling districts the rates varied from 0 to 5.38%. Many factors could be responsible for the variability, such as the H5N1 infection rate and vaccination rate of the poultry, the precautions and job duties of the workers. In our study, poultry number was identified as a novel risk factor associated with human infection with avian H5N1 virus, which could be accounted for the distinction of seropositive rate among districts and studies.

Poultry workers were expected to have the highest level of exposure to avian H5N1virus and more intensive poultry exposure was associated with having anti-H5N1 antibody [11]. Compared with poultry workers from large scale poultry farms, backyard workers may have an even higher risk due to the lack of preventive measures and healthy environment. Wang et al. conducted a serologic surveillance study in Guangzhou, China, founding workers in scale poultry farms all seronegative for H5N1 virus [9]. Certain job duties such as butchering poultry, feeding poultry and preparing poultry for restaurants may also be associated with the infection risk [11]. In our study, enrolled poultry workers were all from backyard poultry farms. Their job duties were almost identical, mainly including feeding poultry, collecting eggs and cleaning poultry stalls.

High poultry density could raise the infection risk of poultry with avian influenza H5N1. Tiensin et al. conducted an ecologic investigation on risk factors of clusters of avian H5N1 virus infection. Fighting cock flock density, meat and laying duck flock density were found significantly higher in case subdistricts than in control subdistricts [12]. However, the association between increasing poultry number and risk of humans infected with avian influenza H5N1 was identified for the first time in this study. This finding could help public health officers recognize high-risk population and institute countermeasures.

Because up to 40% of the poultry workers enrolled in our study were ≥60 years old and the microneutralization and western blot assays were found to be less specific for persons ≥60 years old [5], the horse RBC HI assay, which has high reliability and good agreement with MN assay results without age limit was used in this study [7].

Our findings suggested that avian-to-human transmission of influenza H5N1 virus remains low in China. Workers associated with raising larger poultry flocks have a higher risk of seropositivity. There were several potential reasons for that, such as high poultry density could raise the infection risk of poultry with avian influenza H5N1 [12], and more intensive poultry exposure was associated with having anti-H5N1 antibody for poultry workers [11]. As we didn't know the infection rate of the poultry and the moderate sample size limited the statistical significance of our research, further studies are warranted to validate our results.

## Conclusions

Avian-to -human transmission of avian H5N1 virus remained low. Workers associated with raising larger poultry flocks have a higher risk of seropositivity.

## Competing interests

The authors declare that they have no competing interests.

## Authors' contributions

XH conceived of the study, participated in its design and coordination, performed the statistical analysis and draft the manuscript. RZ participated in the conception, design and coordination of the study, and helped to draft the manuscript. XQ, YQ carried out the Hemagglutination inhibition (HI) assay. LL, ZH helped to draft the manuscript. FT, FZ participated in the design and coordination of the study. All authors read and approved the final manuscript.

## Pre-publication history

The pre-publication history for this paper can be accessed here:

http://www.biomedcentral.com/1471-2334/12/93/prepub

## References

[B1] World Health Organization Avian influenza update http://www.wpro.who.int/health_topics/avian_influenza/Accessed 11 January 2012

[B2] Wang H Feng Z Shu Y Yu H Zhou L Zu R Huai Y Dong J Bao C Wen L Wang H Yang P Zhao W Dong L Zhou M Liao Q Yang H Wang M Lu X Shi Z Wang W Gu L Zhu F Li Q Yin W Yang W Li D Uyeki TM Wang Y Probable limited person-to-person transmission of highly pathogenic avian influenza A (H5N1) virus in China Lancet 2008 371 1427 1434 10.1016/S0140-6736(08)60493-6 18400288

[B3] Li FC Choi BC Sly T Pak AW Finding the real case-fatality rate of H5N1 avian influenza J Epidemiol Community Health 2008 62 555 559 10.1136/jech.2007.064030 18477756

[B4] Briand S Fukuda K Avian influenza A (H5N1) virus and 2 fundamental questions J Infect Dis 2009 199 1717 1719 10.1086/599209 19416077

[B5] Rowe T Abernathy RA Hu-Primmer J Thompson WW Lu X Lim W Fukuda K Cox NJ Katz JM Detection of antibody to avian influenza A (H5N1) virus in human serum by using a combination of serologic assays J Clin Microbiol 1999 37 937 943 10074505 10.1128/jcm.37.4.937-943.1999 PMC88628

[B6] Guo Y Li J Cheng X Discovery of men infected by avian influenza A (H9N2) virus Zhonghua Shi Yan He Lin Chuang Bing Du Xue Za Zhi 1999 13 105 108 12569771

[B7] Kayali G Setterquist SF Capuano AW Myers KP Gill JS Gray GC Testing human sera for antibodies against avian influenza viruses: horse RBC hemagglutination inhibition vs. microneutralization assays J Clin Virol 2008 43 73 78 10.1016/j.jcv.2008.04.013 18571465 PMC2574547

[B8] World Health Organization Recommendations and laboratory procedures for detection of avian influenza A (H5N1) virus in specimens from suspected human cases http://www.who.int/csr/disease/avian influenza/guidelines/RecAIlabtestsAug07.pdf

[B9] Wang M Fu CX Zheng BJ Antibodies against H5 and H9 avian influenza among poultry workers in China N Engl J Med 2009 360 2583 2584 10.1056/NEJMc0900358 19516044

[B10] Ortiz JR Katz MA Mahmoud MN Ahmed S Bawa SI Farnon EC Sarki MB Nasidi A Ado MS Yahaya AH Joannis TM Akpan RS Vertefeuille J Achenbach J Breiman RF Katz JM Uyeki TM Wali SS Lack of evidence of avian-to-human transmission of avian influenza A (H5N1) virus among poultry workers, Kano, Nigeria, 2006 J Infect Dis 2007 196 1685 1691 10.1086/522158 18008254

[B11] Bridges CB Lim W Hu-Primmer J Sims L Fukuda K Mak KH Rowe T Thompson WW Conn L Lu X Cox NJ Katz JM Risk of influenza A (H5N1) infection among poultry workers, Hong Kong, 1997-1998 J Infect Dis 2002 185 1005 1010 10.1086/340044 11930308

[B12] Tiensin T Ahmed SS Rojanasthien S Songserm T Ratanakorn P Chaichoun K Kalpravidh W Wongkasemjit S Patchimasiri T Chanachai K Thanapongtham W Chotinan S Stegeman A Nielen M Ecologic risk factor investigation of clusters of avian influenza A (H5N1) virus infection in Thailand J Infect Dis 2009 199 1735 1743 10.1086/599207 19416075

